# Fear of Sexual Harassment Accusations: A Hidden Barrier to Opposite-Gender Mentoring in Taiwan?

**DOI:** 10.3390/bs14020137

**Published:** 2024-02-15

**Authors:** Thomas R. Tudor, Stephanie D. Gapud, Naeem Bajwa

**Affiliations:** 1 School of Business, University of Arkansas at Little Rock, Little Rock, AR 72204, USA; 2 Division of Business, Spring Hill College, Mobile, AL 36608, USA

**Keywords:** sexual harassment, mentoring, accusation fears, social exchange theory, career advancement

## Abstract

While legal protections against sexual harassment are crucial, their implementation could have unintended consequences. This study explores the potential downside of these protections—fear of false accusations—and its impact on cross-gender mentoring in Taiwanese workplaces. Drawing on social exchange theory, we investigate how fear of accusations might discourage valuable mentoring relationships between men and women. Through an intercept survey, we examined whether these concerns may lead to reduced mentoring opportunities for women, potentially hindering their career advancement. We proposed new constructs and analyzed the model using SmartPLS 4.1. Our findings reveal a complex dynamic: fear of accusations does appear to decrease cross-gender mentoring, raising concerns about its impact on women’s career trajectories. However, the findings also suggest that men support sexual harassment laws, still believing these laws are needed. We discuss our model and its implications; additionally, we emphasize the need for strategies that balance legal protections while also fostering positive mentoring relationships.

## 1. Introduction

Two main general types of sexual harassment are legally recognized in Taiwan [1]. The first is often referred to globally as quid pro quo, which involves a negation action experienced for sexual compliance refusal [2,3,4,5,6,7,8,9,10]. The second is often called a hostile sexual work environment [2,6,11,12], which involves sexual behavior that “has the purpose or effect of unreasonably interfering with an individual’s work performance or creating an intimidating, hostile, or offensive work environment” [13] (p. 1). Sexual harassment does not belong in the workplace, and organizations should follow the law and proactively try to prevent it [14]. But could these needed laws also have unattended consequences?

Could the fear of a possible false accusation of sexual harassment reduce mentoring relationships between men and women? Could this fear, if experienced, make the costs of mentoring another gender not worth the benefits? In addition, if this fear is real and has a negative impact on gender-related mentoring, what could be learned about these fears to help organizations address and reduce them? We do know that quality mentoring relationships are often valued by both employees and organizations [15], as we will discuss in the next section on mentoring. This is particularly true for younger employees who are starting out in their professions [16]. Successful individuals frequently credit one specific or multiple mentors with helping them to succeed in their careers [17,18]. After discussing mentoring, we consider if these accusation fears are conceivably experienced, and if possibly true, we use social exchange theory to try to explain the potential impact of these fears on mentoring. Finally, we use our hypotheses, visualized in our proposed model, to test our assumptions using our collected questionnaire data, along with discussing study outcomes and limitations.

## 2. Mentoring

What is mentoring? “Mentoring is a traditional process in which an experienced person (the mentor) guides another person (the mentee or protégé) in the development of other or his own ideas, learning, and personal/professional competence” [16] (p. 160). Typically, it involves a senior employee helping a younger employee to achieve career progression [19]. “Excellent mentors generally provide two clusters of critical mentoring functions. Psychosocial functions include encouragement, friendship, and emotional support. Career functions include direct teaching, advocacy, coaching, visibility, and challenge” [20] (p. 2). How valuable are mentoring relationships? Xu and Payne [17] assert that it is better for employees to have mentors to support them upon starting new or more experienced roles. Quality mentorships also have a positive impact on job satisfaction and work performance [16,21,22,23,24].

Allen and Eby [25] agree that the mentee is not the only one who benefits, as the organization gains higher-skilled talent, improved productivity, enhanced collaboration, higher motivation, better communication and trust, and higher training cost-efficiency if mentoring works as intended. In other words, successful mentoring improves outcomes for both employees and employers [26]. Past studies have found that those with successful mentoring relationships had higher job satisfaction [27,28] and commitment [17,23,29]; held more positive attitudes about their careers [30]; and were better at obtaining corporate board positions [31].

Is mentoring only valued in Western countries? In the United States, over 70% of Fortune 500 employers offer mentoring schemes to increase productivity and reduce turnover [32]. However, mentoring can also have positive work outcomes in Asian countries. According to a study of 246 employees in Chinese companies, mentoring influences workplace learning and career growth [33]. Moreover, according to a study of 512 workers at a Chinese manufacturing company, mentoring increased work performance and social status [34]. Additionally, in their study of 113 Chinese white-collar workers, Bozionelos and Wang [35] found that those with effective mentors intrinsically believed that this support was beneficial for career success, even when extrinsic outcomes were not always apparent. Moreover, in a recent study of 290 workers from two Chinese firms, mentoring was positively related to worker engagement [36].

What about the value of women being mentored by men? “The research is clear: women in competitive, historically male, “up-or-out” organizational cultures make more money and enjoy more rapid promotions when men mentor them” [20] (p. 2). Unfortunately, women have reported that it is harder to find supportive male mentors, even though men are more likely to be in higher-status positions, enabling them to provide mentoring services [20,31,37,38]. This is particularly true for informal mentoring [39]. Some men might find mentoring women challenging because of concerns about office gossip or negative perceptions. Even if unfounded, these concerns could contribute to gender disparities in mentorship opportunities [40].

## 3. Gender Concerns and Possible Impact

Many men are concerned that the frequent personal contact required for mentoring could be interpreted as sexual when mentoring women, and as a result, they fear hurting their public image [41]. Men have reported not wanting to work alone with women for fear of being accused of sexual harassment or sexual assault [42,43]. Indeed, when mentoring women, men are less likely to give tough professional criticism relative to similar feedback given to other men, denying women improvement opportunities [20]. This is unfortunate because women are typically unrepresented in higher management, meaning that they could benefit the most from proper mentoring by men, using the resulting feedback to achieve greater success [15,44,45]. In addition, women lacking useful mentors may experience reduced self-confidence and self-efficacy, as well as miss opportunities to establish important professional networks and increase their interpersonal skills [41]. Reviewing 50 years of scholarship involving women, Flores, Settles, McGillen, and Davis [46] assert that research is lacking regarding how the global #MeToo movement has changed mentoring between men and women. One useful theory for investigating how mentoring might have changed between men and women is social exchange theory (SET), one of the theories most widely used in behavioral research to explain reciprocal relationships between individuals [47].

## 4. Theory and Hypothesis

Social exchange theory posits that an employee’s belief in the worthiness of a relationship with another employee is connected to their inclination to form this relationship [48]. Using social exchange theory, mentoring is thought to be reciprocal, with both parties gaining personal and/or professional benefits [44,45,49]. These mutual benefits can lead to valued relationships, particularly with individuals who are trusted because sharing, as required for effective mentoring, makes people vulnerable [50,51]. In addition, individuals can purposely decrease physical [52] and emotional interactions with individuals if a threat is perceived [53]. This can be amplified because employees are often uncomfortable with asking for help [54,55,56], and help is often needed by those with better capabilities and knowledge [57,58] for mentoring.

The mentor and mentee must both benefit for the mentoring scheme to be effective [59]. More specifically, the mentor must achieve satisfaction from the relationship, such as a feeling of fulfillment [51,60]. Previous studies have mostly focused on what the mentee might gain, neglecting the equally important psychological needs of the mentor [61,62]. Although mentoring can be important and even critical for the mentee’s success, the mentor must also benefit in some way for it to be successful, and they should not view mentoring as a “costly burden” [63] (p. 604). Thus, our first hypothesis makes the following prediction:

**H1.**  *Fears of being accused of workplace sexual harassment are positively related to diminished mentoring between colleagues of different genders*.

These fears of accusation are also assumed to be related to the perception that sexual harassment laws have not fully benefited women as intended. This perception does not mean that those with fears of accusation want workplace sexual harassment to be legal and do not care about victims of sexual harassment, as these questions were not asked or implied. However, based on social exchange theory, people with such higher fears are thought to experience them because of these laws, and they may react by reducing their interactions with women. It seems reasonable that those with these fears would have similar perceptions on whether these laws have fully benefited women. Thus, our second hypothesis makes the following prediction:

**H2.**  *Fears of being accused of workplace sexual harassment accusation are related to the perception that sexual harassment laws have benefited the careers of women.*

This fear is believed to be felt mostly by men, as the perceived risk of mentoring women is increased with the possibility of a future sexual or gender harassment allegation [64]. For women, Bergen and Bessler [41] assert that commendable efforts to create gender equality because of #MeToo and Time’s Up have encouraged male fears of unfairness, leading to the “adoption of behavior and attitudes opposite of the intended effects…” (p. 19). A harassment claim could destroy a man’s reputation and career, with social media helping to magnify this issue [65]. Respondents to a Pew Research survey reported strong concerns about men being dismissed for sexual harassment without hearing all the facts, with 51% agreeing that men today have difficulty knowing how to appropriately interact with women in the workplace [66]. Even sexual harassment training is believed to increase awkwardness in male–female interactions and male fears of facing accusations [67]. Thus, our next two hypotheses make the following predictions:

**H3.**  *Fears of being accused of workplace sexual harassment are impacted by gender.*

**H4.**  *Gender is related to diminished gender mentoring.*

It is also reasonable to assume that men are more likely to view sexual harassment laws as not fully benefiting women even if they support these laws, whereas women, as the main benefactor, would be more likely to have an opposing view. However, women may also recognize the limits to these laws in relation to their careers, as sexual harassment has not been eliminated. Thus, our next hypothesis makes the following prediction:

**H5.**  *Gender is related to the variability in the perception that sexual harassment laws have benefited the careers of women.*

**Figure 1:**
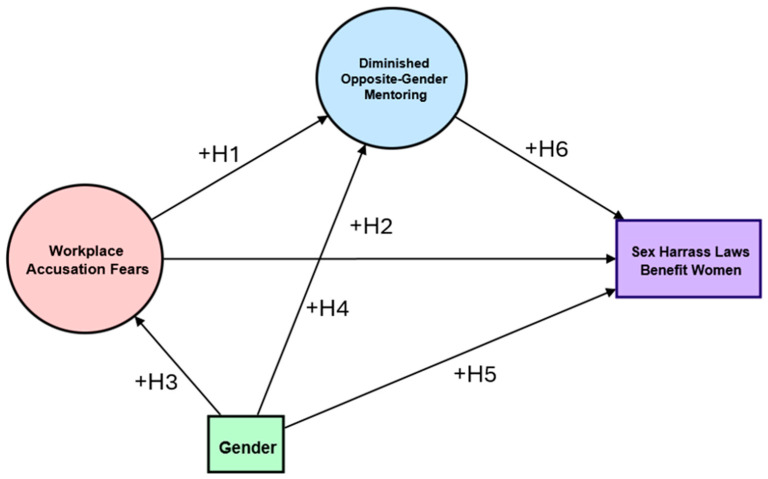
Simplified model showing the hypothesized relationships.

Finally, a reduction in opposite-gender mentoring is likely related to sexual harassment legislation. In social exchange theory, the risks versus rewards for mentoring are thought to be influenced by these laws, along with a reduction in the value of the reciprocal relationship. Thus, our last hypothesis is shown below, and our proposed model is shown in Figure 1.

**H6.**  *Diminished opposite-gender mentoring is related to the perception that sexual harassment laws have benefited the careers of women.*

## 5. Research Method

A brief paper survey was created using original questions to fit our study as no other suitable questions were available from other research. The questions were translated from English into Mandarin Chinese using a professional translation service that performed forward translation and back translation to double-check the language’s meaning accuracy. The survey was reviewed again by a native Mandarin Chinese speaker with no further change recommendations and pretested in Taiwan to check the understanding of the survey questions. No problems were found, so the survey was used.

Data were collected from subjects walking near major workplaces who were at least 18 years old. Subjects were asked to complete a short, anonymous survey on workplace sexual harassment. A five-response Likert scale was utilized for these questions, using strongly agree, somewhat agree, neither agree nor disagree, somewhat disagree, and strongly disagree. The survey subjects were welcome to change their minds and choose not to complete it. The survey was designed to take 2 to 3 min maximum to increase response rates and minimize fatigue during completion. A survey with 321 respondents was completed by men (44%) and women (56%). Their ages were mixed, with 18 to 22 (9%), 22 to 35 (29%), 31 to 40 (29%), 41 to 50 (23%), 51 to 65 (9%), and over 65 (1%). Their marital statuses consisted of single (43%), married (40%), divorced/separated (16%), and widow (1%). Their employers ranged in size from under 25 employees (26%), 26 to 50 employees (30%), 51 to 100 employees (25%), to over 100 (18%).

## 6. Data Analysis and Results

**Figure 2:**
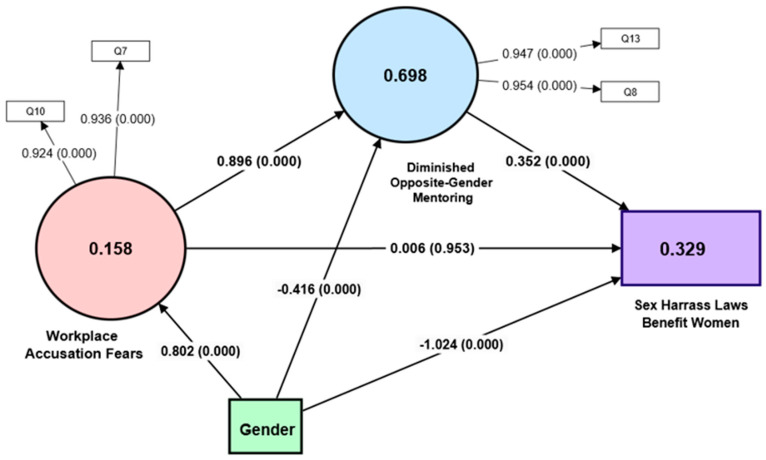
The measurement model shows the path coefficients. The numbers in parentheses are the *p*-values, and the numbers inside the dependent variables are the R^2^. Note: Workplace Accusation Fears and Diminished Opposite-Gender Mentoring are latent constructs made up of several variables, while Sexual Harassment Laws Benefit Women and Gender are single-item variables.

We utilized partial least square structural equation modeling (PLS-SEM) using SmartPLS software version 4.1 to examine the theoretical relationships present in the collected sexual harassment data [68]. PLS-SEM is a multivariate technique that combines aspects of factor analysis and multiple regression to simultaneously examine inter-related dependence relationships between the measured variables and latent constructs (variates), as well as between latent constructs [69]. We used this technique because of the flexibility it offers when analyzing the interplay between theories and actual data for theory development [70]. PLS-SEM uses proxies to represent the constructs of interest, which are weighted composites of indicator variables for a particular construct. Thus, our research using PLS-SEM explains both the total variance and the common variance [71]. Our structural equation model was assessed using a two-step approach. The first step was to analyze the measurement model, as shown in Figure 2, with a description of the measures shown in Table 1. Using the method of Hair et al. [69,71], the rules for model assessment and sample size were applied. The second step was the appropriate analysis of the structural model [70,72,73].

### Measurement Confirmation

As seen in Figure 2, we propose Workplace Accusation Fears and Diminished Opposite-Gender Mentoring as constructs. Workplace Accusation Fears relate to general fears of being accused of sexual harassment. Diminished Opposite-Gender Mentoring relates to specific sexual harassment accusation fears that have reduced or even prevented strong mentoring relationships with colleagues of the opposite gender.

We evaluated the construct and convergent validity to assess their accuracy. Construct validity is the extent to which a set of measured items reflects the theoretical latent construct that those items are designed to measure [69]. Convergent validity occurs when items that are indicators of a specific construct share a high proportion of common variance. Examining the size of factor loadings in Figure 2 indicates that all variables are loaded to their respective construct at a higher rate than the 0.702 minimum thresholds [69].

Next, we examined the average variance extracted (AVE). There is adequate convergence of the variables if the AVE of the construct is higher than 0.50. If the AVE is less than 0.50, on average, more error remains in the items than the variance explained by the latent factor structure imposed on the measure. The AVEs of Workplace Accusation Fears (0.865) and Diminished Opposite-Gender Mentoring (0.903) are greater than the minimum threshold of 0.5, suggesting that the variables that make up these constructs exhibit convergence validity.

Composite reliability (CR) was also examined. High construct reliability indicates that internal consistency exists, meaning the items are highly correlated and measure the same thing. Construct reliability, as a measure of convergent validity, assesses whether the items measuring a construct consistently represent the same underlying concept [69]. All the reflective constructs in the model exceeded the minimum requirement of 0.70 for composite reliability. Moreover, the rule of thumb for construct reliability is that Cronbach’s alpha and composite reliability should be greater than 0.7. As seen in Table 2, all reliability measures (Diminished Opposite-Gender Mentoring, 0.893, and Workplace Accusation Fears, 0.844) are above the 0.7 threshold. This means that the variables that make up these two constructs exhibit high reliability.

*Discriminant validity* is the extent to which a construct is truly distinct from other constructs. In other words, high discriminant validity is evidence that a construct is unique and captures some phenomena that other measures do not capture. There are two measures of discriminant validity reported in the SmartPLS 4.1 analysis results: the Fornell–Larcker criterion and the heterotrait–monotrait (HTMT) criterion. Using the Fornell–Larcker criterion, discriminant validity for the two constructs in our model is achieved. According to this criterion, the square root of the average variance extracted by a construct (Diminished Opposite-Gender Mentoring, 0.95; Workplace Accusation Fears, 0.93) must be greater than the correlation between the construct and any other construct (0.894). However, recent literature suggests that the HTMT criterion is the better criterion to determine discriminant validity [69,70,74]. HTMT is defined as the mean value of the item correlations across constructs relative to the (geometric) mean of the average correlations for the items measuring the same construct. Our two constructs are theoretically similar; therefore, we expect that there is a high HTMT score between them [75]. This is because the concepts of fear and sexual harassment are included in each construct’s measures. In Table 3, the two constructs have a 0.933 discriminant validity score. This score is just a little above the 0.90 threshold [70,74]. However, we will proceed with the structural analysis because this is an exploratory study. Furthermore, if collinearity is a concern, there is another measure that will be discussed in the following section starting in Table 4.

## 7. Assessment of the Structural Model

Structural model coefficients for the relationships between the constructs are derived by estimating a series of regression equations. Collinearity between constructs must be examined to make sure it does not bias the regression results. To measure the increase in regression coefficient due to collinearity, a variance inflation factor (VIF) analysis was performed for each set of predictor constructs in the model. VIF values above five are indicative of probable collinearity issues among predictor constructs [69]. As seen in Table 3, the VIF between Workplace Accusation Fears and Diminished Opposite-Gender Mentoring (1.187) is less than the lower threshold of three and way below five; therefore, there is no critical collinearity between them [69,72,74,75,76]. Our results show that all the VIFs of constructs in the inner model and the variables in the outer model were above 0.20 and below 5.0, which indicates that the model does not exhibit collinearity problems [74,77].

### 7.1. Test of Hypothesis

We employed bootstrapping to evaluate the significance of hypothesized relationships between dependent and independent variables in our model. This method enabled us to estimate *p*-values and confidence intervals, enhancing the reliability and generalizability of our findings. Most hypothesized relationships achieved statistical significance, confirming their expected impact. However, the second hypothesis, examining the path between “Workplace Accusation Fears” and “Sexual Harassment Laws Benefit Women”, did not. This implies that fear of being accused of sexual harassment alone does not directly influence the perception of women’s promotions. Indirectly, however, its effect on the dependent variable is explained by the mediator variable Diminished Opposite-Gender Mentoring (Table 5).

### 7.2. In-Sample Prediction Results

To evaluate the underlying theory of the model, hypothesis tests, R^2^ values, mediation analysis, IPMA, and predictive relevance were examined. Table 5 provides analyses of path coefficients and hypothesis tests. All hypothesized relationships were statistically significant except for the relationship between workplace accusation fears and sexual harassment laws that benefit women.

The coefficient of the determination (R^2^ evaluation) of the variables measures the predictive power of the model. Table 6 below lists the R^2^ interpretation values. There is a huge variation between disciplines in the interpretation of R^2^ values. Cohen [78] proposed a classification system for interpreting R^2^ values in psychology. He categorized values of 0.0196, 0.1304, and 0.2592 as representing small, medium, and large effect sizes, respectively. Marketing has a much higher requirement for R^2^ values, with 0.75, 0.50, and 0.25, respectively, described as substantial, moderate, and weak [79]. The dependent variable Sexual Harassment Laws Benefit Women has a large coefficient of determination (0.329) using Cohen’s rule of thumb [78]. Diminished Opposite-Gender Mentoring (0.698) has large predictive accuracy (R^2^) when using the classifications set by Cohen [78]. For psychology research results, this number is only moderately high. This indicates that almost 70% of the variation in “Diminished Opposite-Gender Mentoring” is explained by the predictors (Workplace Accusation Fears and Gender) in the model.

Workplace Accusation Fears as a construct is being predicted as weak-to-medium strength by Gender. This could mean that there are other variables that impact this construct that are not specified in the model. Moreover, perceptions of Sexual Harassment Laws Benefit Women are strongly predicted by the model. This suggests that the independent variables in the model (Diminished Opposite-Gender Mentoring, Workplace Accusation Fears, and Gender) play a significant role in shaping these perceptions.

The within-sample fit effect size (F-square (*f*^2^), as shown in Table 7, also known as the explained variance or goodness-of-fit, is a measure of how well a statistical model explains the observed data. It is assessed by calculating the F-square (*f*^2^) value of the model. F-square is considered an in-sample predictive metric. An *f*^2^ value of 0.02 is considered to be a small effect size, an *f*^2^ value of 0.15 is considered to be a medium effect size, and an *f*^2^ value of 0.35 is considered to be a large effect size [78]. Gender has a medium-sized effect (0.115–0.288) on all the variables it is connected to in the model. Diminished Opposite-Gender Mentoring has a small effect on Sexual Harassment Laws Benefit Women. Workplace Accusation Fears have a very large effect (2.242) on Diminished Opposite Gender Mentoring. This is interesting because this means that fear of being accused of sexual harassment is largely affecting the mentoring opportunities that women can obtain from men who occupy leadership positions in the organization.

## 8. Importance–Performance Map Analysis (IPMA)

**Figure 3:**
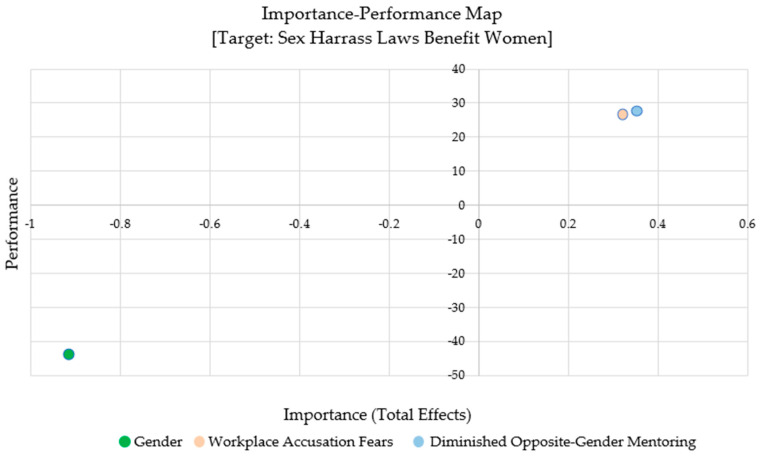
IPMA [Target: Sexual Harassment Laws Benefit Women].

First, we checked that all the outer measures in our model were loaded positively. Then, we analyzed our data using the IPMA available in SmartPLS. The IPMA allows for prioritizing constructs to improve a certain target construct. Our purpose is to first identify which predecessor construct or variable in the model has the most impact on sexual harassment laws that benefit women. Figure 3 shows that Diminished Opposite-Gender Mentoring has a 0.352 effect on Sexual Harassment Laws Benefit Women at a rate of 27.648%. Figure 3, therefore, shows that any improvement in the mentoring of women in the workplace will strongly impact the perception that Sexual Harassment Laws Benefit Women in the workplace. Additionally, Diminished Opposite-Gender Mentoring has a positive importance value of 0.352. This indicates that a lack of mentoring opportunities from individuals of the opposite gender may hinder women’s career progression.

## 9. Discussion

### 9.1. Unraveling the Complexities: Fear, Mentoring, and Gender

Social exchange theory posits that individuals engage in interactions based on perceived costs and benefits. In the workplace, this translates to employees making decisions about effort, loyalty, and engagement based on their perceived rewards and risks [48,50]. While Table 5 shows that the initial direct effect of “Workplace Accusation Fears” on “Sex Harass Laws Benefit Women” is not statistically significant [H2], this is because a mediating factor called “Diminished Opposite-Gender Mentoring” explains the relationship, as also shown in Table 5, when looking at the statistically significant indirect effects. This fear, primarily impacting men, might outweigh the perceived mentoring benefits (career help for the mentee), leading to diminished engagement in these relationships. This disproportionately harms women, particularly those seeking male mentors, who may miss out on valuable guidance and support. This dynamic disrupts the expected balance of social exchange. Men might fear potential accusations and reputational damage, leading to mentoring hesitancy driven by self-interest.

### 9.2. The Ripple Effect: Reduced Mentoring and the Glass Ceiling

This reduced mentoring may further exacerbate the glass ceiling. Women may lose out on critical career advice, networking opportunities, and sponsorship from senior colleagues, hindering their advancement [80]. The negative relationship between gender and willingness to mentor individuals of the opposite gender aligns with social exchange theory. Perceived risks associated with potential accusations might lead to avoidance of interaction with female colleagues, isolating women and possibly limiting their career development.

### 9.3. Bridging the Gap: Leveraging Social Exchange for Change

Organizations can utilize social exchange theory to design mentoring programs that emphasize reciprocal benefits. Highlighting the advantages for both mentors and mentees, irrespective of gender, may encourage individuals to engage in mentoring relationships, contributing to the dismantling of the glass ceiling. Additionally, transparent communication about the positive impact of sexual harassment laws for everyone may foster a collective belief in the equitable exchange of benefits and break down gender-related barriers.

### 9.4. Gender Perceptions of Sexual Harassment Laws

The findings highlight how gender can influence perceptions of costs and benefits associated with sexual harassment laws. While the findings suggest that men still support these laws and feel they are needed, men who view these laws as increasing their risk of false accusations may perceive them as a cost too, potentially leading to avoiding close interactions with female colleagues. This may isolate women and may limit their opportunities for career development. The findings reveal a gendered perspective on the perceived full benefits of sexual harassment laws, aligning with social exchange theory’s emphasis on individual perceptions of fairness. Gender significantly influences how individuals perceive all the advantages of these laws. Organizations, therefore, should focus on promoting a culture of gender equality and fairness. This includes transparent communication about the positive impact of sexual harassment laws for everyone, fostering a collective belief in the equitable exchange of benefits and contributing to the breakdown of gender-related barriers.

## 10. Conclusions

This research underscores the need to extend our focus beyond mere compliance with sexual harassment laws. It emphasizes the critical importance of delving into the nuanced reactions and apprehensions individuals, particularly men, may harbor because of the fear of accusations. The hesitancy to engage in mentoring relationships with women, stemming from this fear, emerges as a significant barrier that may detrimentally impact the career trajectories of women in the workplace.

Furthermore, the findings of this research offer valuable insights into the intricate dynamics involving gender, workplace apprehensions about accusations, mentoring relationships, and the perceptions of sexual harassment laws. By illuminating these complex interactions, the study not only identifies challenges but also points toward the necessity for proactive interventions. There seems to be a need for organizations to implement measures that transcend the confines of legal compliance.

Proactive initiatives can address the multifaceted challenges posed by gender dynamics, workplace fears of accusations, and the complexities surrounding mentoring relationships. By doing so, organizations can actively contribute to the creation of more inclusive and supportive workplaces, particularly for women. In essence, this research suggests there is a need for change. With a better understanding of the issues and by addressing them, organizations can likely create more equitable workplaces where mentorship flourishes and individuals, irrespective of gender, can thrive in their professional journeys.

## 11. Managerial Implications

Organizations should evaluate sexual harassment complaint procedures to ensure transparency and fairness, conduct confidential surveys to gauge employee perceptions of fairness, and implement positive changes based on feedback. In addition, they should implement formalized mentoring programs that pair experienced individuals with mentees for guidance, support, and career development. These programs can offer valuable opportunities for both genders. To incentivize participation, they should consider offering mentors rewards like monetary compensation, recognition programs, or development opportunities. This can help address potential concerns and encourage engagement.

Organizations should encourage open discussions about concerns surrounding mentor–mentee relationships, especially those related to potential sexual harassment accusations. Furthermore, they should facilitate safe spaces for mentors and mentees to voice concerns and seek guidance, encourage men to mentor women without fear of unfounded accusations, promote a culture where both genders can benefit from mentorship opportunities, and track the progress and outcomes of implemented initiatives. They should assess their impact on employee perceptions, career advancement, and overall organizational culture; be prepared to adapt and refine strategies based on ongoing data and feedback; and cultivate a culture of continuous improvement, actively seeking input from employees and stakeholders to ensure long-term success in promoting gender equality and fostering effective mentoring relationships. By embracing these strategies, managers can create a workplace where women can thrive and reach their full potential.

## 12. Limitations of the Study and Future Research Directions

While this study breaks new ground by exploring the impact of sexual accusation fears on gender-related mentoring in Taiwan (and appears to be one of the first studies on this topic globally), it has limitations that should inform future research directions. As a pioneer study, its design is necessarily exploratory, albeit theoretically grounded in social exchange theory. Future research could benefit from designing longitudinal tests that establish causal relationships and assess the long-term impact of mentoring on women’s career progression. While the sample size was satisfactory, its cross-sectional nature and non-random sampling from a single city limit the data’s generalizability to the entire Taiwanese workforce [81].

Future studies could employ random sampling and include participants from diverse regions and industries for greater representativeness. In addition, survey-based data, while valuable, has inherent limitations. Future research could incorporate open-ended questions, interviews, or mixed-method approaches to gain a richer understanding of participants’ experiences and perspectives. Finally, socio-cultural factors unique to Taiwan may influence the study’s findings. Future research could explore how these fears and their impacts on mentoring dynamics might vary between cultural contexts.

## Figures and Tables

**Table 1:** Descriptions of the measures used in this analysis.

Variable Name	Survey Question	Survey Question
Workplace Accusation Fears	Q10	If single, the fear of a potential sexual harassment complaint would discourage you from dating in your workplace.
	Q7	You personally fear being falsely accused of sexual harassment.
Diminished Opposite-Gender Mentoring	Q13	Mentoring between men and women has diminished because of fear among men of facing a potential sexual harassment complaint.
	Q8	The fear of a potential sexual harassment complaint has prevented you from forming a strong mentoring relationship with a member of the opposite sex.
Sexual Harassment Laws Benefit Women	Q15	Women today are more likely to be promoted and reach higher-level positions because of the protection provided by sexual harassment laws.
Gender	Q20	Gender is coded as male = 2, female = 1

**Table 2:** Construct reliability and validity.

	Cronbach’s Alpha	Composite Reliability (rho_a)	Composite Reliability (rho_c)	Average Variance Extracted (AVE)
Diminished Opposite-Gender Mentoring	0.893	0.896	0.949	0.903
Workplace Accusation Fears	0.844	0.848	0.928	0.865

**Table 3:** Discriminant validity assessment using the heterotrait–monotrait (HTMT) criterion.

	Diminished Opposite-Gender Mentoring	Gender	Sexual Harassment Laws Benefit Women	Workplace Accusation Fears
Diminished Opposite-Gender Mentoring				
Gender	0.157			
Sexual Harassment Laws Benefit Women	0.300	0.452		
Workplace Accusation Fears	0.933	0.435	0.098	

Note: The threshold for the HTMT criterion is 0.90 [76].

**Table 4:** Collinearity statistics; variance inflation factor (VIF) values.

Outer Model VIF Values		Inner Model (VIF) Values			
Variables	VIF	Construct/Variables in the Model	Diminished Opposite-Gender Mentoring	Gender	Sexual Harassment Laws Benefit Women
Q20	1.0	**Gender**			1.328
Q8	2.860	**Diminished Opposite-Gender Mentoring**		1.187	3.316
Q13	2.860				
Q7	2.143	**Sexual Harassment Laws Benefit Women**			
Q10	2.143				
Q15	1.0	**Workplace Accusation Fears**	1.187	1.000	3.849

**Table 5:** Results of Hypothesis Testing.

	Path Coefficients	Standard Deviation (STDEV)	T Statistics (|O/STDEV|)	*p*-Values	Hypothesis Decision
Workplace Accusation Fears ⟶ Diminished Opposite-Gender Mentoring	0.896	0.023	39.629	<0.001	H1: Accept
Workplace Accusation Fears ⟶ Sexual Harassment Laws Benefit Women	0.006	0.107	0.059	0.953	H2: Reject
Gender ⟶ Workplace Accusation Fears	0.802	0.092	8.755	<0.001	H3: Accept
Gender ⟶ Diminished Opposite-Gender Mentoring	−0.416	0.072	5.807	<0.001	H4: Accept
Gender ⟶ Sexual Harassment Laws Benefit Women	−1.024	0.117	8.751	<0.001	H5: Accept
Diminished Opposite-Gender Mentoring ⟶ Sexual Harassment Laws Benefit Women	0.352	0.099	3.561	<0.001	H6: Accept
**Indirect Effects**					
Gender ⟶ Diminished Opposite-Gender Mentoring ⟶ Sexual Harassment Laws Benefit Women	−0.147	0.044	3.346	0.001	
Workplace Accusation Fears ⟶ Diminished Opposite-Gender Mentoring ⟶ Sexual Harassment Laws Benefit Women	0.316	0.09	3.509	<0.001	
Gender ⟶ Workplace Accusation Fears ⟶ Diminished Opposite-Gender Mentoring ⟶ Sexual Harassment Laws Benefit Women	0.253	0.08	3.146	0.002	
Gender ⟶ Workplace Accusation Fears ⟶ Diminished Opposite-Gender _Mentoring	0.719	0.091	7.888	<0.001	
Gender ⟶ Workplace Accusation Fears ⟶ Sexual Harassment Laws Benefit Women	0.005	0.087	0.059	0.953	

**Table 6:** R^2^ Values of variables in the path model.

	R-Square	R-Square Adjusted	Psychology Rules of Thumb for R-Square	Marketing Rules of Thumb
Diminished Opposite-Gender Mentoring	0.698	0.697	Large	Moderately high
Workplace Accusation Fears	0.158	0.155	Medium	Weak
Sexual Harassment Laws Benefit Women	0.329	0.322	Large	Between weak and moderate

Note: Cohen [78] classified the strength of the predictive accuracy (R^2^ values) as either small (0.0196), medium (0.1304), or large (0.2592).

**Table 7:** Within-sample fit effect size: *f*^2^ values of variables.

	*f* ^2^
Diminished Opposite-Gender Mentoring ⟶ Sexual Harassment Laws Benefit Women	0.056
Gender ⟶ Diminished Opposite-Gender Mentoring	0.119
Gender ⟶ Sexual Harassment Laws Benefit Women	0.288
Gender ⟶ Workplace Accusation Fears	0.187
Workplace Accusation Fears ⟶ Diminished Opposite-Gender Mentoring	2.242
Workplace Accusation Fears ⟶ Sexual Harassment Laws Benefit Women	0.001

Note: Rules of thumb for assessing *f*^2^ values follow Cohen [78]—small (0.02), medium (0.15), and large (0.35).

## Data Availability

The data for this study are available from the corresponding author upon request.
